# Prey size preference in the tokay gecko (*Gekko gecko*)

**DOI:** 10.1163/1568539X-bja10251

**Published:** 2023-11-13

**Authors:** Anja Probst, Eva Ringler, Birgit Szabo

**Affiliations:** Division of Behavioural Ecology, Institute of Ecology and Evolution, University of Bern, 3032 Bern, Switzerland

**Keywords:** cognition, foraging, reptile, spontaneous quantity discrimination, Squamata

## Abstract

The optimal foraging theory posits that animals aim to maximize energy intake while minimizing predation and handling costs during foraging. Most observed animal behaviour supports this theory, but occasional deviations provide insights into the ecological factors that shape foraging decisions. We tested prey-size preference using a two-choice test between different prey sizes in tokay geckos. We expected geckos to prefer larger prey and decision latencies to be longer when discrimination was more difficult and when small prey was offered. Geckos preferred larger prey when the size difference was large, although decision latency remained consistent. This aligns with prior research on sit-and-wait predators. Together with previous findings showing freezing behaviour after prey capture in tokay geckos, our findings suggest a strong influence of predator avoidance on foraging decisions opening up a new avenue for future research investigating the link between decision making and predator avoidance in tokay geckos.

## 1 Introduction

The optimal foraging theory predicts that animals should select food items that maximize the profit per unit handling time (MacArthur & Pianka, 1966). Prey should, therefore, not be attacked if the effort for handling and/or the risk of being predated while handling are too high (Ille, 1991; Lemos-Costa et al., 2016). Consequently, a positive relationship between the risk an animal is willing to take in subduing a prey and their need for food is expected (Ille, 1991). The optimal foraging theory, however, does not account for all possible factors that might affect foraging decisions (Emlen, 1966). An animal’s ecology, behaviour, morphological traits, and life history as well as prey availability may cause variation across individuals in the prey they choose (Perry & Pianka, 1997; Bolnick et al., 2003).

Prey size is one important factors when it comes to choosing prey (Griffiths, 1980). Chimpanzees (*Pan troglodytes*), for example, prefer large quality prey, but they avoid prey that is too large to be safely captured (Bugir et al., 2021). Similarly, barn owls (*Tyto alba*) prefer large prey even though profitability decreases with increasing size due to increased handling time (Ille, 1991). Furthermore, guppies tested on their choice between two food patches differing in item size but not amount of food preferred larger food items (Lucon-Xiccato et al., 2015). Not all animals, however, prefer to hunt only large prey. Grey seal (*Halichoerus grypus*), for example, choose prey size in relation to the specific area they hunt in. They hunt for small prey in the depth and for large prey at the surface. This way they have enough time to return to the surface to breathe (Brown & Mate, 1983). Furthermore, some species even prefer medium or small prey (e.g., Brodie & Formanowicz, 1983; Kumaraguru et al., 2011). Therefore, choice behaviour might follow what is predicted by the optimal foraging theory like in the example from Chimpanzees mentioned above (Bugir et al., 2021) while in other cases additional factors might change choice behaviour to seemingly suboptimal prey choice (Perry & Pianka, 1997; Shafir & Roughgarden, 1998).

Only a few studies investigated the prey size related choice behaviour in lizards (Shafir & Roughgarden, 1998). Anoles (*Anolis gingivinus*), for example, prefer relatively large over relatively small prey (Shafir & Roughgarden, 1998) and adult, male Eastern fence lizards (*Sceloporus undulatus*) also prefer large prey while juvenile females preferred smaller prey (Eberhart & Ruby, 2019). Italian wall lizards (*Podarcis siculus*) also preferred larger insect larvae (size) in a quantity discrimination test but failed to choose the larger number of single prey items (Miletto Petrazzini et al., 2017). By contrast, Gidgee skinks (*Egernia stokesii*) choose the larger amount of food, but only when the quantity differed in number not when quantities differed in size (Szabo et al., 2021). Some lizard species, however, show a preference towards a certain prey size, not necessarily large prey (Pérez-Mellado et al., 1991; Diaz, 1995; Chen & Jiang, 2006). Species differ in a number of traits including foraging mode (active versus sit-and-wait) and the type of food they consume (insectivorous versus omnivorous; Cooper, 1995). It has been hypothesised that sit-and-wait predators prefer larger food items compared to active foragers to minimise predation risk and maximise energy gain when encounters are infrequent (Andrews, 1979; Purwandana et al., 2016). Consequently, information on a broader range of lizard species with a range of foraging strategies will improve our understanding of how different ecological conditions and life-history traits impact on foraging strategies and prey preferences.

The aim of this study was to investigate prey size preference of the tokay gecko (*Gekko gecko*), a large (up to 185 mm snout vent length), arboreal and nocturnal (Grossmann, 2006), sit-and-wait predator (Cooper, 1995) that feeds mainly on insects and sometimes on small vertebrates (e.g., Bucol & Alcala, 2013). The hunting and prey-capture behaviour of the tokay gecko is very spontaneous and unpredictable with a freezing phase (characterized by inactivity) often following the capture of a prey (Montuelle & Williams, 2015). As insectivorous sit-and-wait predators, we expected tokay geckos to prefer larger prey when given the choice between two prey items of different sizes (Andrews, 1979; Shafir & Roughgarden, 1998; Purwandana et al., 2016; Miletto Petrazzini et al., 2017). We hypothesised, however, that discriminability would decrease if prey items were more similar in size (Miletto Petrazzini et al., 2017; Szabo et al., 2021). Finally, we also expected to find longer latency to attack when the two presented prey were more similar as a sign for increased processing time to maintain accuracy (Chittka et al., 2009) but expected shorter latencies when attacking larger prey as a sign of higher motivation to capture more profitable, large prey.

## 2 Methods

### 2.1 Animals, captive conditions and husbandry

In this study, we tested 22 captive bred, adult tokay geckos (*Gekko gecko*), 10 males (SVL: mean = 14.77, SD = 0.64, range = 14.06−15.80 cm; weight: mean = 112.4, SD = 11.26, range = 95−131) and 12 females (SVL: mean = 13.37, SD = 0.60, range = 12.49−14.34 cm; weight: mean = 98.42, SD = 15.28, range = 82−135) from different breeders, with an age of approximately 2−6 years. Sex of individuals was determined by the presence (male) or absence (female) of femoral glands (Grossmann, 2006).

At the time of the study, most individuals were kept in pairs in rigid foam terraria (90 L × 45 B × 100 H cm), two females and one male were kept singly (females: 45 L × 45 B × 70 H cm; male: 90 L × 45 B × 100 H cm). At our facility, enclosures are set-up on shelves (small enclosures on the top, large enclosures on the bottom) across two rooms. The ground of the enclosures consists of a drainage layer of expanded clay topped with organic rainforest soil (Dragon BIO-Ground). To prevent mixing, the two layers are separated with a mosquito mesh. We add autoclaved red oak leaves and sphagnum moss on top of the soil to provide food and shelter for isopods and earthworms that break down the faecal matter produced by the geckos (bioactive setup). Enclosures are also equipped with a compressed cork back wall, cork branch tubes cut in half (refuges hanging on the back wall) as well as solid cork branches and life plants as enrichment. We provide a heat mat (Tropic Shop) on the outside of each enclosure that increases the local temperature by 4−5°C for thermoregulation.

Animals are kept in a fully controlled environment with a reversed 12 h:12 h photoperiod (light: 6 pm to 6 am, dark: 6 am to 6 pm) enabling us to work with the animals during their active period (night). A dim red light (PHILIPS TL-D 36 W/15 RED) not visible to the geckos (Loew, 1994) is kept on for 24 h and provides some visibility within animal rooms. The automatic system simulates sunrise and sunset which are accompanied by a gradual change in temperature which reaches from approximately 30°C during the day cycle and 25°C during the night cycle. Additionally, enclosures are equipped with a UVB light on top (Exo Terra Reptile UVB 100, 25 W). The humidity is kept at 50% and daily rainfall (reverse osmotic water, 30 s every 12 h at 5 pm and 4 am) increases the humidity to 100% for a short period of time to simulate natural tropical conditions.

Geckos are fed three times per week on Monday, Wednesday, and Friday with 3−5 adult house crickets (*Acheta domesticus*). Crickets are fed with cricket mix (reptile planet LDT), Purina Beyond Nature’s Protein™ Adult dry cat food, and fresh carrots for optimal nutrition (Vitamin D and calcium). Lizards are fed with 25 cm long forceps to monitor their food intake and have a water bowl that provides water ad libitum. The lizards are weighed once a month and measured form snout to vent every two months to monitor their health.

### 2.2 Testing procedure

To reduce stress, we tested the lizards in their home enclosure (Langkilde & Shine, 2006). In the first step of the testing procedure, a dim white light (LED, SPYLUX® LEDVANCE 3000 K, 0.3 W, 17 lm) was put on top of the enclosure (mesh lid) for better visibility and to be able to video record gecko behaviour. Geckos expect food or testing every time this light occurs. Thereafter, the focal lizard was located and if it was behind a refuge, the refuge was carefully removed. Next, the experimenter (always the same person) prepared two 25 cm long forceps in their right hand and pinched a live house cricket of different size by its’ head (to ensure that the body was visible during the presentation) in each forceps to present to the lizard. Before each presentation the experimenter ensured that both crickets were 5 cm apart using a ruler. Then, the focal individual was approached with both forceps to a distance of approximately 4 cm from its snout (optimal attack distance, personal observation) while ensuring that both crickets were presented equidistant from the lizard’s snout. This procedure was chosen because lizards were used to feed from tweezers and to be able to test them within their familiar home enclosure. The animal was then allowed to choose one cricket to attack and consume. A trial lasted for a maximum of 60 s and the gecko’s behaviour was recorded on video (iPhone 13 pro, 12 megapixels) to be scored later. Trials in which an individual did not choose a cricket within 60 s were not repeated (22% or *N* = 171 of 792 valid trials). Geckos were tested on feeding days only (Monday, Wednesday, or Friday). The order in which the lizards were tested was randomized each test day. Data were collected from 2 March and 4 May 2022 between approximately 8:15 am and 12:00 pm.

### 2.3 Stimuli

Geckos were tested with three different cricket-size combinations: (1) a medium cricket and a small size cricket (MvS), (2) a large cricket and a small cricket (LvS), and (3) a large cricket and a medium cricket (LvM). Each test day an individual received one session of each prey combination (one trial each = three trials) for a total of 12 sessions presenting each cricket-size combination only once per session. The order in which the three different combinations were presented was pseudo-randomized and counterbalanced. This ensured that the side the smaller cricket was presented occurred no more than twice per combination. All crickets were alive during presentation and we made sure that they were moving to attract the geckos’ attention.

### 2.4 Prey selection

**Figure 1:**
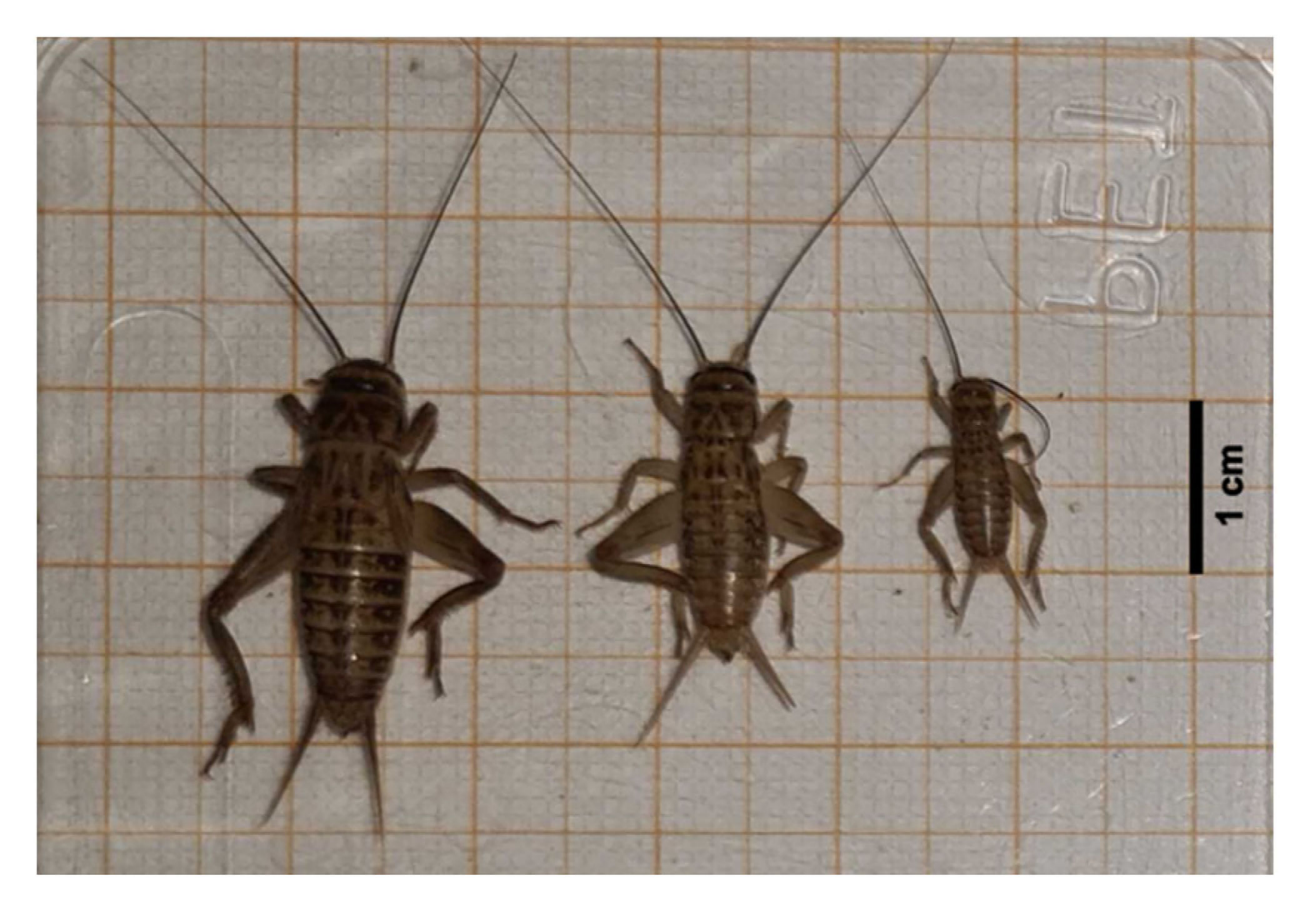
Example of each cricket’s size used during the experiment. Small crickets were 1.0 ± 0.1 cm, medium crickets were 1.5 ± 0.1 cm and large crickets were 2.3 ± 0.1 cm.

Crickets were selected from our breeding population and sorted into three size categories. Ahead of the experiment, we randomly picked 10 crickets of the categories small, medium, and large each based on visual judgment and measured their body length with callipers. The mean ±0.1 cm of the 10 crickets per size category was then used to classify the different size categories. Crickets were defined as small when they were 1.0 ± 0.1 cm, medium when they were 1.5 ± 0.1 cm and large when they were 2.3 ± 0.1 cm. As these size categories were easy to distinguish visually (Figure 1) and abundantly present, on test days, crickets were selected based on visual judgment.

### 2.5 Data collection

To determine if lizards showed a preference for a cricket size, we recorded which size of cricket the focal individual chose first during trials. Additionally, we measured the time from when a gecko first noticed the two prey objects (by turning its’ head to focus on the prey) until it attacked (attack latency) from the videos. Using BORIS, the free behavioural coding software (Friard & Gamba, 2016), we measure the attack latency to 0.001 s by slowing down the videos to half their speed. If a gecko did not attack a prey within 60 s, the attack time and choice was recorded as NA leading to some lizards receiving less than 12 trials for some of the three prey combinations (range_LvM_ = 1−10; range_LvS_ = 2−12; range_MvS_ = 1−10).

### 2.6 Statistical analyses

To investigate if geckos preferred to attack larger prey, we ran a Bayesian linear mixed model (GLMM, package MCMCglmm; Hadfield, 2010) with family Binomial using the larger choice (yes = 1 and no = 0, Bernoulli variable) as the response variable and the prey combination (MvS, LvS or LvM) as a fixed effect. Additionally, we included sex and the scaled mass index (SMI, Peig & Green, 2009) as fixed effects. The scaled mass index was calculated as follows:

$$\text{SMI}=M_{i}{\left[\frac{L_{0}}{L_{i}}\right]}^{b_{\text{SMA}}}$$

where *M*_*i*_ is the weight of each individual *i, L*_*i*_ the SVL of each individual *i, L*_0_ is the mean weight of the study population and *b*_SMA_ is the scaling exponent estimated by the standardised major axis (SMA) regression of weight and SVL. To be able to compare prey combinations we used Post Hoc least square means comparisons (EMM, package emmeans; Lenth, 2021) and results are reported on the log-scale.

We were also interested if the time taken to attack (attack latency) was influenced by the prey combination or the size of the prey. We ran another Bayesian linear mixed model with family Gaussian using the log-transformed latency as the response variable and the prey combination (MvS, LvS or LvM) as well as prey size category (S, M, or L) as fixed effects. Again, we included sex and the scaled mass index as additional fixed effects. Latency was log-transformed to better fit a Gaussian distribution. Both models included a random intercept of animal identity and a random slope of trial nested in session to account for repeated testing, differences in the number of trials across individuals and autocorrelation across successive samples.

Finally, we also investigated if geckos were biased towards left or right when choosing prey. We, therefore, recorded choice as either to the right (=1) or left (=0) prey presented during each trial and ran a two-sided binomial test with the number of choices to the right compared to all valid choices.

For all Bayesian models we made sure that no autocorrelation occurred (correlation between lags <0.1 Hadfield, 2010), that the MCMC chain sufficiently mixed (by visually inspecting plots, Hadfield, 2010) and was run long enough (Heidelberg and Welch diagnostic tests, Hadfield, 2010). All analyses were run in R version 4.0.3 (R Development Core Team, 2022). Where possible, we report results as *p* > 0.1 no evidence, 0.1 < *p* < 0.05 weak evidence, 0.05 < *p* < 0.01 moderate evidence, 0.01 < *p* < 0.001 strong evidence, *p* < 0.001 very strong evidence (Muff et al., 2022) or as evidence when the Higher Posterior Density Interval did not cross 0. All data and code generated during this study are available on the Open Science Frame-work (doi: 10.17605/OSF.IO/CQD59).

### 2.7 Ethical note

We followed the guidelines provided by the Association for the Study of Animal Behaviour and the Animal Behaviour Society for the treatment of animals in behavioural research and Teaching (ASAB Ethical Committee & ABS Animal Care Committee, 2023). Tests were non-invasive and lizards were not forced to participate. All animals stayed at our research facility after the experiment was finished to participate in future experiments. The experiment was approved by the Suisse Federal Food Safety and Veterinary Office (National No. 33232, Cantonal No. BE144/2020). Captive conditions were approved by the Suisse Federal Food Safety and Veterinary Office (Laboratory animal husbandry license: No. BE4/11).

## 3 Results

**Figure 2:**
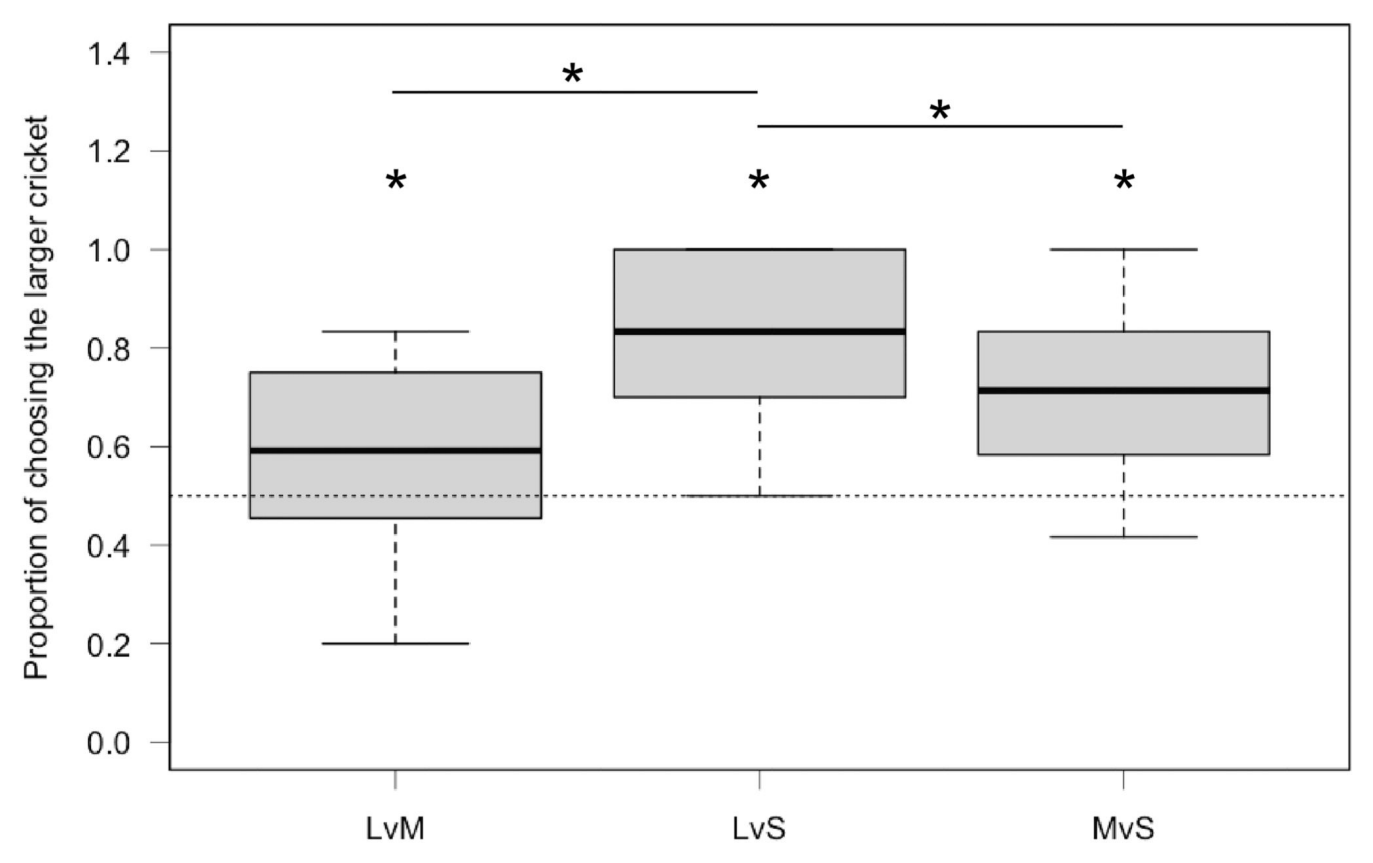
Boxplots of the proportion of choices made towards the larger cricket across the three presented combinations: LvM, large versus medium; LvS, large versus small; MvS, medium versus small. The dotted line represents random choice of 50% (0.5). The bold line within the boxes shows the median, the upper box edges show the upper quartile, the lower box edges the lower quartile, the top whisker ends show the maximum and the bottom whisker ends the minimum (outliers are not shown). *p < 0.05.

We found evidence that geckos chose the larger prey above chance in all three combinations but chose the larger cricket less often (less accurate) when choosing between large and medium sized prey (EMM, mean = 0.542, CI_low_ = 0.137, CI_up_ = 0.922, Table 1), more accurate when choosing between medium and small sized prey (EMM, mean = 0.951, CI_low_ = 0.546, CI_up_ = 1.332, Table 1) and most accurate when choosing between large and small sized prey (EMM, mean = 1.918, CI_low_ = 1.484, CI_up_ = 2.404; Figure 2, Table 1).

We found evidence that lizards were more accurate choosing the larger prey when presented with large and small prey compared to both large and medium sized prey (EMM, estimate = −1.382, CI_low_ = −1.945, CI_up_ = −0.871) and medium and small sized prey (EMM, estimate = 0.963, CI_low_ = 0.436, CI_up_ = 1.543; Figure 2). We only found weak evidence that geckos were also more accurate when choosing between medium and small sized prey compared to large and medium sized prey (EMM, estimate = −0.407, CI_low_ = −0.900, CI_up_ = 0.097).

We found no evidence that accuracy (choosing the larger prey) differed between males and females (GLMM, estimate = −0.471, CI_low_ = −1.024, CI_up_ = 0.127, *p* = 0.114) but found weak evidence that individuals in better condition (higher SMI) were marginally more accurate (GLMM, estimate = 0.020, CI_low_ = −0.002, CI_up_ = 0.042, *p* = 0.082).

**Figure 3:**
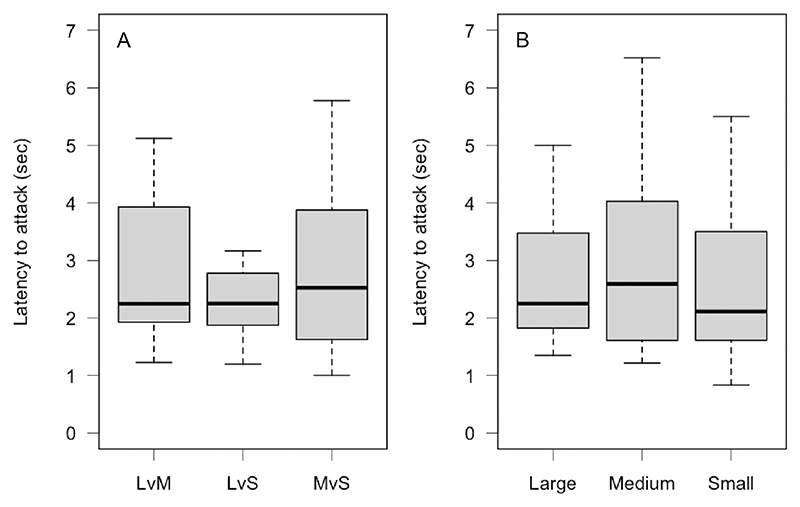
Boxplots of the latency to attack the chosen cricket. (A) Across the three presented combinations: LvM, large versus medium; LvS, large versus small; MvS, medium versus small. (B) Across the three presented prey sizes. The bold line within the boxes shows the median, the upper box edges show the upper quartile, the lower box edges the lower quartile, the top whisker ends show the maximum and the bottom whisker ends the minimum (outliers are not shown).

We found no evidence that attack latency differed across the three presented prey combinations (GLMM, estimate_LS_ = 0.030, CI_low_ = −0.196, CI_up_ =0.247, *p* = 0.794; estimate_MS_ = 0.134, CI_low_ = −0.108, CI_up_ =0.396, *p* = 0.294, reference level MvS; Figure 3A), that lizards attack latency differed across prey sizes (GLMM, estimate_M_ = 0.003, CI_low_ = −0.254, CI_up_ =0.258, *p* = 0.972; estimate_S_ = −0.013, CI_low_ = −0.304, CI_up_ =0.277, *p* = 0.936, reference level L; Figure 3B), that attack latency differed between males and females (GLMM, estimate = −0.140, CI_low_ = −0.425, CI_up_ =0.142, *p* = 0.331) or that it was related to body condition (GLMM, estimate = −0.008, CI_low_ = −0.018, CI_up_ =0.002, *p* = 0.139).

Finally, we found no evidence that geckos exhibited side preferences during the trial except for one lizard which showed weak evidence to prefer crickets presented on the right side (binomial test, choices to the right side = 24, number of trials = 36, probability of success = 0.667, CI_low_ = 0.490, CI_up_ =0.814, *p* = 0.065; Table 1) even though the smaller crickets were presented on the right side 18 out of 36 trials.

## 4 Discussion

Our results demonstrate that tokay geckos prefer larger over smaller prey when given the choice between two prey items differing in size. The accuracy of choosing the larger prey was related to the presented combination. Lizards were most accurate (chose the larger prey more often) when choosing between large and small sized prey compared to the other two combinations (large and medium, medium and small) which is in line with our prediction that more similar prey sizes were harder to discriminate. Contrary to accuracy, we found no relationship between prey size and attack speed and we found no evidence that lizards exhibited a side bias when choosing.

Our results confirm our expectation that tokay geckos, similar to other sit-and-wait predators (e.g., Shafir & Roughgarden, 1998; Eberhart & Ruby, 2019), prefer larger prey items. For insectivorous lizards, such as the tokay gecko, energetic costs of prey capture are negligible compared to the energy gain from insects as they provide high energy levels (Pough & Andrews, 1985; Cruz-Neto et al., 2001) although net energy gain still decreases with increasing prey size (Pough & Andrews, 1985). Furthermore, tokay geckos are able to chew large prey items and process them into smaller pieces ready to swallow (Shine & Thomas, 2005). Additionally, previous work has shown that tokay geckos prefer prey that can be captured in a single event (Andrews & Bertram, 1997). Finally, tokay geckos often show a freezing phase after prey capture (Montuelle & Williams, 2015). Together, this suggests that they likely choose large prey to reduce hunting time (Andrews & Bertram, 1997) while avoiding being spotted by predators (Andrews, 1979). In our experiment we did not present prey that was overly large but well within a size range that could be easily handled and swallowed (predation on small vertebrates: Bucol & Alcala, 2013; Krysko & Love, 2016). There might be a limit when, due to large prey size, prey handing becomes too costly either due to increased handling effort (chewing) and increasing conspicuousness to predators while handling prey and/ or increased escape probability if the prey is not killed on first strike. It would be interesting to investigate the maximum prey size that is still attacked and the relationship between processing time and prey size (Andrews & Bertram, 1997). Furthermore, if tokay geckos prefer large prey items in the wild, what are their limits in prey size and what are naturally occurring prey size needs to be determined in future studies.

We also predicted that the accuracy to choose the larger prey item would decrease when the offered prey sizes were more similar, which made comparisons more difficult. Our results are in accordance with this prediction. It was easier for geckos to distinguish small from large crickets compared to small and medium or medium and large crickets. This is further supported by our results that geckos were equally likely to choose the larger prey in combination medium-large and small-medium. Similar preferences were found across different reptile species. Italian wall lizards (*P. sicula*) preferred the larger insect larvae (Miletto Petrazzini et al., 2017). Similarly, Herman’s tortoises (*Testudo hermanni*) prefer to choose larger tomato slices (Gazzola et al., 2018). One limitation of our study is that we only used prey from three size categories which prevents us from making any inferences about the limits in discriminatory abilities. Tokay geckos rely predominantly on vision to hunt, a common characteristic of sit-and-wait lizard predators (Cooper, 1995). It would, therefore, be interesting to extend our methodology to include a wider range of prey sizes to really understand geckos’ ability to distinguish prey size on a finer scale. Furthermore, from our experiment, we are unable to determine what information geckos used to choose the larger prey (e.g., size, volume, movement). As prey was still alive during presentation one possible cue apart from size could have been movement if one prey size moved more during presentation. Salamanders choose the larger quantity of food based on movement (Krusche et al., 2010) and, as visual hunters, geckos might have also been influenced by prey movement but if this was the case should be investigated in future studies. Interestingly, we found a weak relationship between body condition and accuracy to choose the larger prey. Geckos in better body conditions were more accurate in choosing larger prey. If being in better body condition made lizard more accurate or if more accurate lizards were able to keep in better body conditions (by choosing larger prey overall) cannot be determined from our study.

Contrary to our expectations, attack latency was not related to the prey combination or prey size. We expected that lizards would attack large prey faster to secure them (motivation) and because they are easier to capture (larger bodies to bite into). Attack latency did not change no matter what prey size was attacked. The geckos are accustomed to feed on prey presented in forceps. They might have learnt that prey presented in such a way is not able to flee making it unnecessary to change attack latency. Additionally, attack latency might increase (processing time) to maintain accuracy when a discrimination is hard (Chittka et al., 2009). The fact that we were unable to detect differences in attack latency could be due to our test being too simple by providing easily discriminable prey sizes and only two options. We could decrease the ratio and increase the number of options presented to increase task difficulty and investigate if this leads to the expected differences in processing time (Chittka et al., 2009). Additionally, in future experiments, trial time could be limited to put more pressure on lizards to make a decision possibly affecting latency in trials with decreased discriminability.

Finally, we did not find a difference in prey size preference between the sexes even though males and females were kept in pairs for breeding and all females were producing eggs. Differences in prey preference between the sexes can be related to differences in energetic needs and time budgets especially during the mating season when males tend to look for mates while females need energy for gestation/ incubation (Schoener, 1971). An observational study in wild tokay geckos found similar results (Aowphol et al., 2006) although studies in other lizards have found differences between males and females (e.g., Preest, 1994). Rather than a shift in size preference, male and female tokay geckos might shift to prefer other prey species differing in nutrient content which could be investigated during and outside the breeding season especially in wild individuals.

In summary our study reveals prey size preferences in captive bred tokay geckos. Our analysis shows that geckos prefer large prey, that males and females do not differ in their prey size preference and that attack latency is not related to prey size. Tokay geckos foraging behaviour is in line with that of other insectivorous sit-and-wait lizard predators (Shafir & Roughgarden, 1998; Grossmann, 2004). Tokay geckos’ preference for large prey, prey they can capture in a single attack (Andrews & Bertram, 1997) and a distinct freezing phase after prey capture (Montuelle & Williams, 2015) indicate that, predator avoidance seems to have a strong influence on foraging behaviour in this species. Future studies should investigate the minimum difference in prey size that tokay geckos can still distinguish, to better understand how precise geckos are in their prey choice.

## Figures and Tables

**Table 1:** Individual number of right side choices during trials, total number of choices (i.e., number of valid trials), number of time the smaller crickets was presented on the right side, proportion of choices to the right side and *p*-value based on a two-sided binomial test.

Individual identity (and sex)	Number of right side choices	Total choices	Number of time the small prey was on the right	Proportion of right side choices	*p*	LvM	LvS	MvS
G001 (♀)	18	36	18	0.50	1.00	0.750	0.833	0.583
G002 (♀)	22	36	18	0.61	0.24	0.417	0.833	0.750
G003 (♂)	12	18	6	0.67	0.24	0.333	1.000	1.000
G004 (♂)	22	36	18	0.61	0.24	0.667	0.583	0.417
G005 (♀)	16	35	18	0.46	0.74	0.455	0.833	0.750
G006 (♂)	20	36	18	0.56	0.62	0.833	0.833	0.833
G007 (♀)	16	34	16	0.47	0.86	0.750	1.000	0.727
G008 (♀)	4	8	2	0.50	1.00	0.333	0.667	0.500
G009 (♂)	22	36	18	0.61	0.24	0.750	0.677	0.583
G010 (♀)	13	26	13	0.50	1.00	0.600	1.000	0.556
G011 (♂)	24	36	18	0.67	0.07	0.583	0.667	0.583
G012 (♀)	16	36	18	0.44	0.62	0.750	0.750	0.583
G013 (♂)	19	32	16	0.59	0.38	0.583	0.700	0.700
G014 (♂)	13	19	6	0.68	0.17	0.571	0.500	0.667
G015 (♀)	8	15	3	0.53	1.00	0.200	0.800	0.800
G016 (♀)	16	30	15	0.53	0.86	0.600	1.000	0.900
G017 (♂)	14	32	17	0.44	0.60	0.500	0.910	0.556
G018 (♂)	2	3	0	0.67	1.00	0.500	0.000	1.000
G019 (♀)	8	19	9	0.42	0.65	0.714	1.000	1.000
G020 (♀)	14	26	15	0.54	0.85	0.375	1.000	1.000
G021 (♀)	14	36	18	0.39	0.24	0.667	1.000	0.833
G022 (♂)	20	36	18	0.55	0.62	0.750	0.833	0.417

Additionally, the table includes the proportion of times the larger cricket was chosen in each combination for each individual. LvM, large versus medium; LvS, large versus small; MvS, medium versus small.
